# Sugar and Free Amino Acid Contents in Winter Wheat Flour Under Fusarium Head Blight Treatment and Natural Infection

**DOI:** 10.3390/plants14101504

**Published:** 2025-05-16

**Authors:** Valentina Španić, Beka Sarić, Katarina Šunić Budimir, Jurica Duvnjak, Slađana Žilić

**Affiliations:** 1 Agricultural Institute Osijek, Department of Small Cereal Crops Breeding and Genetics, Južno Predgrađe 17, 31000 Osijek, Croatia; katarina.sunic@poljinos.hr (K.Š.B.); jurica.duvnjak@poljinos.hr (J.D.); 2 Maize Research Institute Zemun Polje, Group of Food Technology and Biochemistry, Slobodana Bajića 1, Zemun, 11185 Belgrade, Serbia; bsaric@mrizp.rs (B.S.); szilic@mrizp.rs (S.Ž.)

**Keywords:** free amino acids, Fusarium, sugars, wheat

## Abstract

Fusarium head blight (FHB) is one of the most devastating diseases in wheat. Besides its negative impact on grain yield, FHB also negatively influences quality. Changes in sugar and free amino acid content were analyzed in flour from *Fusarium*-infected and non-infected grains of six wheat varieties differing in *Fusarium* resistance. The concentrations of sugars and free amino acids were determined using a high-performance liquid chromatography device. In flour from FHB-infected grains, the average total amount of fructose, glucose, maltose, total sugars, and total reducing sugars was significantly increased, compared to non-treated flour from the Tika Taka variety, which was the most FHB-susceptible. The total content of free amino acids in flour from FHB-infected varieties increased in proportion to their susceptibility. In Tika Taka, there was a significant increase in free amino acid content of about 46%, while a significant decrease of 16% was observed in the highly resistant Vulkan variety. A highly significant correlation was established between the degree of FHB susceptibility and the content of aspartic acid, glutamic acid, glutamine and histidine, glycine, alanine, methionine, valine, tryptophan, phenylalanine, leucine, and threonine. Most amino acids had strong positive correlations with each other, but among the sugars, only fructose and glucose content showed a strong positive correlation with specific amino acids that were induced by *Fusarium* infection. Overall, it can be concluded that FHB-susceptible varieties have a high risk of FHB infection, which results in the hydrolysis of sucrose into fructose and glucose, together with an increase in free amino acids, which deteriorates the quality of wheat.

## 1. Introduction

Fusarium head blight (FHB) is a serious fungal disease in wheat (*Triticum aestivum* L.) and other cereals caused by different *Fusarium* species [1]. Besides considerable reductions in grain yield, FHB contaminates grains with mycotoxins and provokes morphological changes in grains and their end-use quality, making wheat flour unsuitable for consumption [2,3,4,5]. The most important *Fusarium* mycotoxins are type A and B trichothecenes and zearalenone [6]. Mycotoxin-contaminated food or feed can result in acute or chronic poisoning of humans and animals. Therefore, the introduction of *Fusarium* mycotoxins poses a significant danger to the food chain and overall food safety. The main causal agent of FHB in wheat is *Fusarium graminearum,* while, occasionally, the disease is caused by other *Fusarium* species, such as *F. culmorum* and *F. avenaceum*, which are also highly representative species [7]. However, some less virulent pathogens, such as *F. poae*, may result in high levels of mycotoxin contamination in infected grains but without visible symptoms [8]. *Fusarium* infection is favored by moist, warm conditions during the flowering stage. The development and intensity of FHB is influenced by host susceptibility and pathogen aggressiveness, but the weather conditions are the most important in disease development [9]. Symptoms of disease may occur over the entire spike or on just a few spikelets, which become bleached white or yellow instead of green. Infection that occurs around flowering results in white grains, so-called Fusarium-damaged kernels, which are shriveled and of low weight. To combat FHB, it is important to integrate plant protection practices such as crop rotation, resistant varieties, and chemical treatments [10]. However, the best method for controlling the disease is the production of wheat varieties resistant to FHB [11,12,13]. FHB severity is a complex trait and is characterized by different resistance types: type I, resistance to initial infection; type II, resistance to the spread of symptoms [14]; type III, resistance to toxin accumulation [15]; type IV, resistance to kernel infection; and type V, yield tolerance [16,17]. As mentioned before, FHB is a global problem in wheat production that causes a considerable loss of grain yield and quality due to reduced protein and starch content. *Fusarium* spp. interferes with the grain hydrolytic protein profile, thereby changing the grain protein content and overall quality [18]. In the case of FHB infection, the fungal mycelium causes a mechanical blocking of the vascular bundle that results in an inadequate supply of grain components [19]. Besides this, *Fusarium* spp. produce enzymes, such as proteases or carbohydrates, which cause changes in the ratio of grain components [20]. For example, proteases alter the gluten network in wheat grains, thus having an impact on the dough’s quality and consequently on bread’s quality and texture [21]. Therefore, changes in carbohydrates and amino acids in grains will also occur, as FHB is a cause of grain biodeterioration, with the fungus being responsible for the production of different hydrolytic enzymes.

This is especially important in the context of the fact that carbohydrates are the most prevalent constituent of wheat grain, forming about 83% of its dry matter [22]. Carbohydrates are a primary product of photosynthesis and have an important role in the synthesis of metabolic compounds and in cell metabolism, the production of energy, stabilization of membranes, and regulation of gene expression [23,24]. The basic units of carbohydrates are simple sugar molecules, such as glucose, maltose, fructose and sucrose, while complex carbohydrates are also known as starch. Sucrose was found to be the major sugar present in the endosperm during early germination, whereas maltose and glucose predominate during the later stage [25]. These simple carbohydrates have the roles of nutrients, hormone-like signaling molecules, and regulators of metabolism, growth, stress responses, and development from embryogenesis to senescence [26]. These molecules belong to the type of simple carbohydrates and have been studied extensively over the years in terms of their structure and functionality, as they are related to a particular end-product of wheat [27]. Starch, a major constituent of the wheat endosperm, plays an important role in determining wheat quality. However, FHB infection will not always influence grain starch and protein content in the same manner, which could be explained by the early build-up of stored proteins during the grain-filling stage [21].

Further, the biological value of a protein is determined by its amino acid profile and the proportions of the content of individual amino acids, and especially, exogenous amino acids (tryptophan, arginine, lysine, histidine, leucine, valine, phenylalanine, and methionine) are very important [28]. The amino acid composition of wheat grain is quite unbalanced, with a lack of essential amino acids like lysine, threonine, and methionine. A decrease in essential amino acid content will further occur after processing wheat grains into various products such as wheat flour [29]. Gardiner et al. [30] analyzed the amino acid content of wheat heads, and it was shown that arginine was a strong deoxynivalenol (DON) inducer, while some other amino acids, such as lysine, methionine, and phenylalanine, were weak DON inducers. In another study by Gardiner et al. [31], some amino acids increased in concentration in wheat heads during FHB disease development, and these included glycine, valine, arginine, alanine, phenylalanine, lysine, and leucine, as well as threonine and/or citrulline. Also, after FHB infection, the concentrations of three aromatic amino acids—phenylalanine, tyrosine, and tryptophan—increased [32]. The exogenous application of amino acids and a *F. graminearum* inoculation assay showed that proline and alanine increased wheat resistance to FHB, while cysteine worsened its susceptibility [33]. During protein hydrolysis under FHB attack, there is also the possibility of the peptide bonds breaking down, which will increase the free amino acid and carboxyl groups [34].

Due to the large consumption of wheat products worldwide, it is of huge importance to understand the impact of *Fusarium* infection on grain and flour properties. The current study focuses on the detection of sugar and free amino acid changes in flour from winter wheat varieties under natural infection and artificial *Fusarium* inoculation.

## 2. Results

### 2.1. General and Type I Resistance

**Figure 1:**
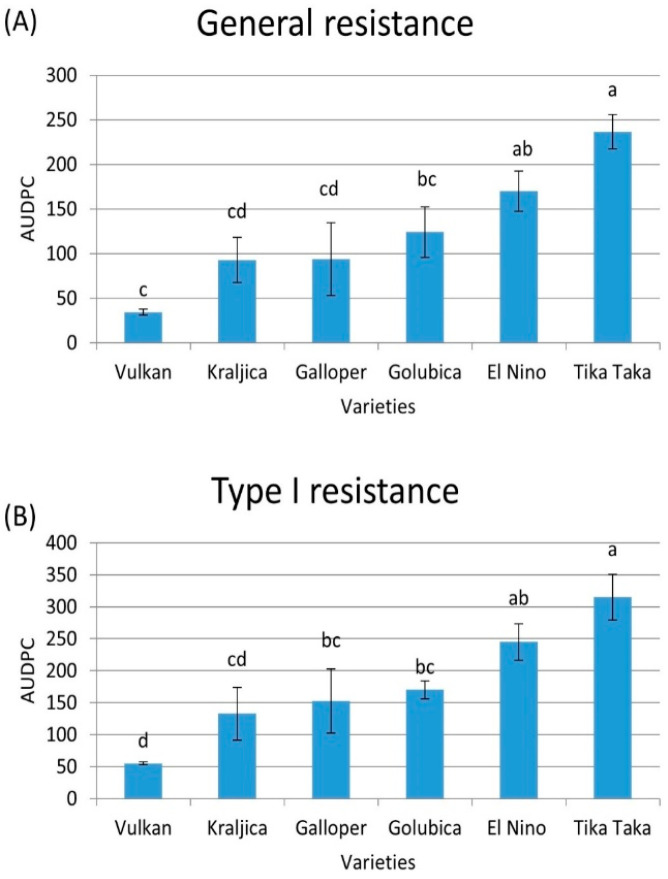
General (**A**) and type I (**B**) resistance in six winter wheat varieties (Vulkan, Kraljica, Galloper, Golubica, El Nino, and Tika Taka) as determined in terms of area under disease progress curve (AUDPC). Bars represent mean values of two independent biological replicates ± SD. Lowercase letters indicate significant difference among varieties (*p* < 0.05).

Figure 1A shows the area under the disease progress curve (AUDPC) value for the general resistance of each variety in FHB-inoculated plants. The Tika Taka variety exhibited the highest disease severity (low general resistance), with an average AUDPC value of 237, followed by El Nino (AUDPC 170) and Golubica (AUDPC 124). The Galloper, Kraljica, and Vulkan genotypes exhibited lower disease severity in comparison to the previous three varieties, with AUDPC values of 94, 93, and 34, respectively.

The highest type I resistance was observed in the Vulkan variety (AUDPC value of 55) in FHB-inoculated plants. An intermediate AUDPC for initial resistance, indicating medium type I resistance, was observed in the Kraljica variety (AUDPC 133), followed by Galloper (AUDPC 153) and Golubica (AUDPC 170). The highest AUDPC for type I resistance (the lowest resistance) was observed for Tika Taka (AUDPC 315), followed by El Nino (AUDPC 245) (Figure 1B). General and type I resistance were not evaluated in control plots as there were no visible symptoms of FHB.

### 2.2. Sugars Under FHB and Naturally Infected Treatment

**Figure 2:**
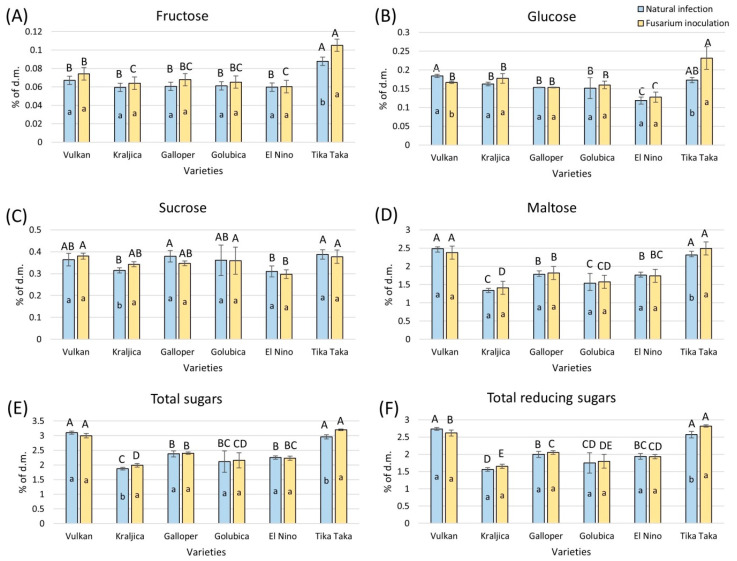
Content of fructose (**A**), glucose (**B**), sucrose (**C**), maltose (**D**), total sugars (**E**), and total reducing sugars (**F**) in flour from six winter wheat genotypes (Vulkan, Kraljica, Galloper, Golubica, El Nino, and Tika Taka) with natural infection and *Fusarium* inoculation. Bars represent means of four independent biological replicates ± SD. Lowercase letters indicate significant differences between treatments in each variety, while capital letters indicate significant differences between varieties in each type of treatment, i.e., natural and artificial infection (*p* < 0.05).

Although there was a slight increase in the content of fructose in flour from most of the varieties artificially infected with FHB compared to naturally infected samples, it was not statistically significant, except in the Tika Taka variety. In flour from this artificially infected variety, this increase in content amounted to 17% (Figure 2A). In both treatments, only Tika Taka showed a significantly higher content of fructose in the flour compared to other varieties.

In FHB-inoculated flour from the Tika Taka variety, glucose significantly increased by 25%, compared to the corresponding naturally infected flour (Figure 2B). The other varieties showed a slight but not significant increase in the content of glucose, except Vulkan, which showed a significant decrease in glucose content. In the naturally infected group, Vulkan showed a significant difference in glucose content compared to Galloper, Golubica, and El Nino, while among artificially FHB-infected flours, Tika Taka was significantly different from the other varieties.

FHB stress did not significantly influence sucrose content in flour from almost all wheat varieties tested (Figure 2C). A significant increase of 8% in sucrose content was observed in artificially FHB-infected flour of the Kraljica variety, compared to naturally infected flour. In naturally infected flour, Tika Taka showed a significantly higher content of sucrose compared to El Nino and Kraljica, while in FHB-treated flour, El Nino had a significantly decreased content of sucrose compared to Vulkan, Golubica, and Tika Taka.

In artificially FHB-infected flour, maltose was not significantly affected in most of the tested varieties (Figure 2D). A significant increase of 7% was only observed in artificially FHB-infected flour of the Tika Taka variety compared to the corresponding naturally infected flour. In flour from both treatments, varieties Tika Taka and Vulkan had a significantly higher maltose content compared to other varieties.

Total sugars significantly increased only in artificially FHB-infected flour from varieties Tika Taka (8%) and Kraljica (6%) compared to naturally infected flour (Figure 2E). In flour from both treatments, varieties Tika Taka and Vulkan had a significantly higher content of total sugars compared to other varieties.

Among FHB-treated flours, the content of total reducing sugars was significantly increased in the Tika Taka variety only, by 9% (Figure 2F). In the naturally infected treatment, varieties Tika Taka and Vulkan had a significantly higher content of total reducing sugars than the rest of the varieties. Among FHB-treated flours, only Tika Taka showed a significantly higher content, followed by Vulkan.

### 2.3. Free Amino Acid Level Under FHB Treatment and Natural Infection

**Figure 3:**
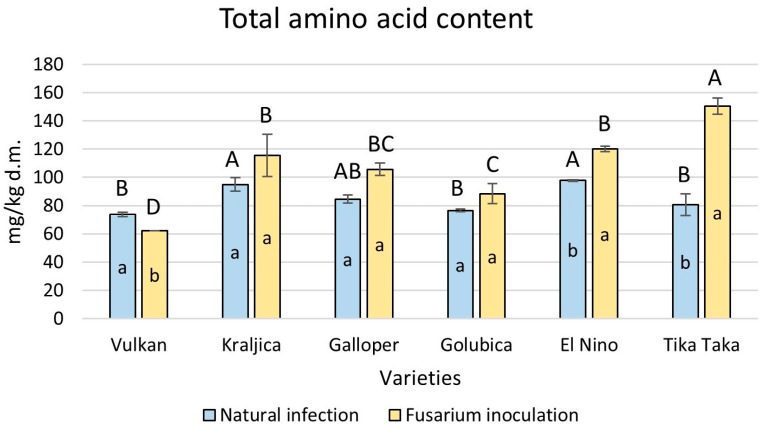
Total amino acid content in flour from six winter wheat genotypes (Vulkan, Kraljica, Galloper, Golubica, El Nino, and Tika Taka) with natural infection and *Fusarium* inoculation. Bars represent means of two independent biological replicates ± SD. Lowercase letters indicate significant differences between treatments in each variety, while capital letters indicate significant differences between varieties in each type of treatment, i.e., natural and artificial infection (*p* < 0.05).

In the wheat flours in each treatment, 15 amino acids in the free state were detected. Among them, aspartic acid, glutamic acid, glycine, alanine, arginine, and asparagine are non-essential amino acids, while glutamine (which is also a non-essential amino acid but could not be properly separated from histidine) plus histidine, methionine, valine, tryptophan, phenylalanine, isoleucine, leucine, and threonine are essential amino acids. Overall, a statistically significant increase in total amino acid content was accomplished by varieties El Nino (19%) and Tika Taka (46%) in flours artificially infected with *Fusarium* compared to naturally infected flours (Figure 3). The highly resistant Vulkan variety had a significant decrease of 16%. It was also evident that Tika Taka had a significantly higher total amino acid content in FHB-treated flour compared to other varieties.

**Figure 4:**
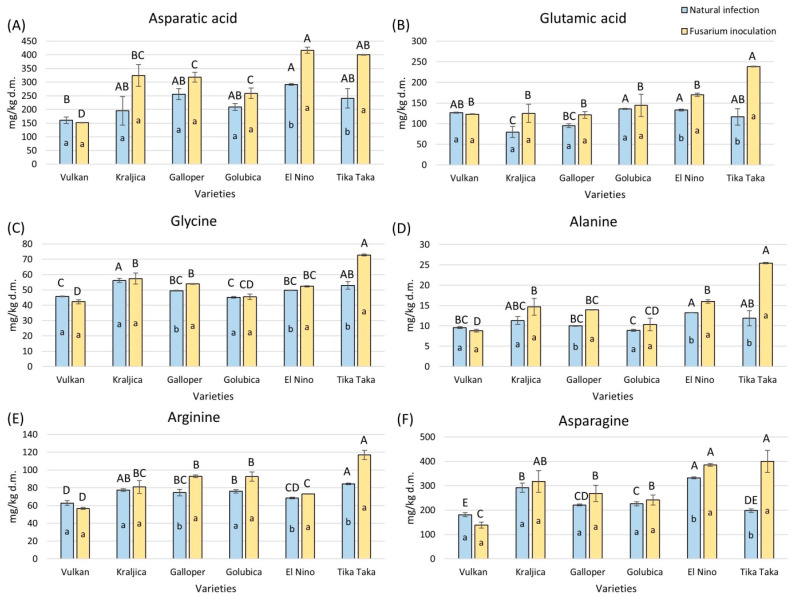
The content of free amino acids—aspartic acid (**A**), glutamic acid (**B**), glycine (**C**), alanine (**D**), arginine (**E**), and asparagine (**F**)—in flour from six winter wheat genotypes (Vulkan, Kraljica, Galloper, Golubica, El Nino, and Tika Taka) with natural infection and *Fusarium* inoculation. Bars represent means of two independent biological replicates ± SD. Lowercase letters indicate significant differences between treatments in each variety, while capital letters indicate significant differences between varieties in each type of treatment, i.e., natural and artificial infection (*p* < 0.05).

Free aspartic acid and free glutamic acid were significantly increased by 40% and 30% in flour from Tika Taka and El Nino wheat plants treated with *Fusarium* spp. (Figure 4A,B). In naturally infected and *Fusarium*-inoculated flour, El Nino had the highest content of free aspartic acid, while glutamic acid showed the highest level in Golubica and El Nino under natural infection and Tika Taka under *Fusarium* inoculation.

In Tika Taka, El Nino, and Galloper, glycine content increased significantly by 27%, 5%, and 8%, respectively, in *Fusarium*-inoculated flour (Figure 4C). In natural infection, Kraljica showed the highest amount of glycine, while in *Fusarium* inoculation, the highest glycine content was detected in Tika Taka.

In flour from *Fusarium* inoculation, Tika Taka, El Nino, and Galloper showed an increase in alanine and arginine, compared to flour from natural infection (Figure 4D,E). The highest increase in alanine and arginine was 53% and 28% for Tika Taka. In natural infection, El Nino had the highest content of alanine, and arginine was the highest in Tika Taka. In *Fusarium* inoculation, Tika Taka had the highest amount of both amino acids, compared to other varieties.

Asparagine was significantly increased in flour from *Fusarium* inoculation compared to natural infection in Tika Taka and El Nino (Figure 4F). The highest increase was about 51% for Tika Taka. El Nino had the highest content of asparagine in natural infection, while both Tika Taka and El Nino showed the highest amount in *Fusarium* inoculation.

**Figure 5:**
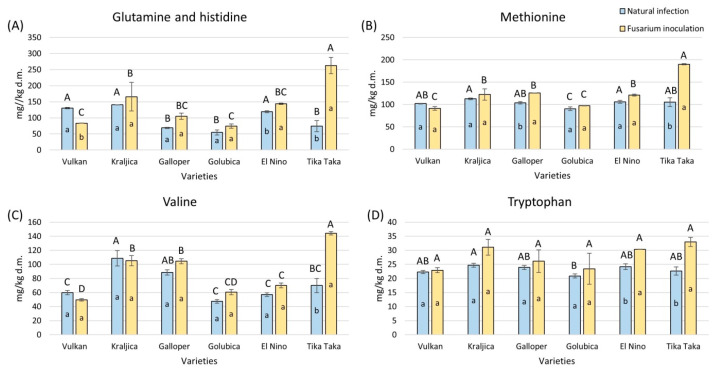
The content of free amino acids—glutamine and histidine (**A**), methionine (**B**), valine (**C**), and tryptophan (**D**)—in flour from six winter wheat genotypes (Vulkan, Kraljica, Galloper, Golubica, El Nino, and Tika Taka) with natural infection and *Fusarium* inoculation. Bars represent mean values of two independent biological replicates ± SD. Lowercase letters indicate significant differences between treatments in each variety, while capital letters indicate significant differences between varieties in each type of treatment, i.e., natural and artificial infection (*p* < 0.05).

The content of glutamine and histidine together was significantly elevated in Tika Taka by 72% and in El Nino by 17% in the flour from *Fusarium* inoculation, while it was significantly decreased in Vulkan (Figure 5A). In natural infection, Vulkan, Kraljica, and El Nino showed the highest amounts of glutamine and histidine, while in *Fusarium* inoculation, this was true for Tika Taka.

Methionine and valine significantly increased by 45% and 51% in Tika Taka in flour under *Fusarium* inoculation, together with methionine in Galloper (Figure 5B,C). In the natural infection group, almost all varieties had a similar content of methionine, except Golubica, which had a significantly lower content. Tika Taka had the highest content of methionine in the *Fusarium* inoculation group compared to other varieties. The highest amount of valine in the natural infection group was obtained for Kraljica and in the *Fusarium* inoculation group for Tika Taka.

Tryptophan significantly increased in flour under *Fusarium* inoculation, compared to natural infection, from Tika Taka and El Nino, by 31% and 20% (Figure 5D). Under natural infection, tryptophan was the highest in Kraljica, while under *Fusarium* inoculation, all varieties had a similar content.

**Figure 6:**
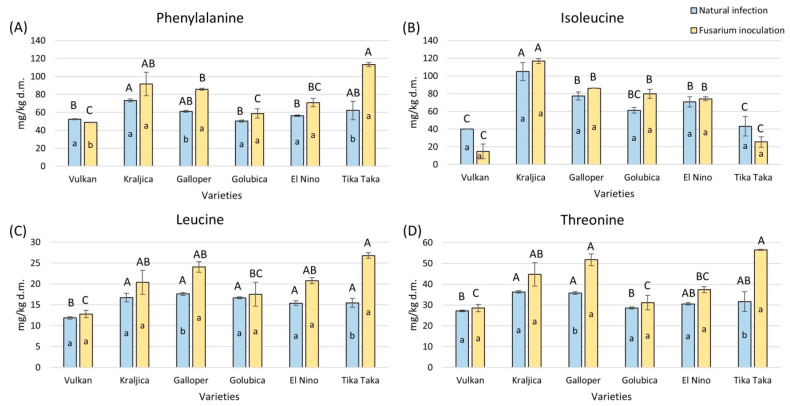
The content of free amino acids—phenylalanine (**A**), isoleucine (**B**), leucine (**C**), and threonine (**D**)—in flour from six winter wheat genotypes (Vulkan, Kraljica, Galloper, Golubica, El Nino, and Tika Taka) with natural infection and *Fusarium* inoculation. Bars represent mean values of two independent biological replicates ± SD. Lowercase letters indicate significant differences between treatments in each variety, while capital letters indicate significant differences between varieties in each type of treatment, i.e., natural and artificial infection (*p* < 0.05).

Tika Taka and Galloper had a significantly increased phenylalanine content in the *Fusarium* inoculation group, while it was significantly decreased in Vulkan (Figure 6A). The highest increase, of 45%, was recorded for Tika Taka. Kraljica had the highest content of phenylalanine under natural infection, while Tika Taka showed the highest amount of phenylalanine under *Fusarium* inoculation.

There were no significant changes in isoleucine in flour from all varieties between the two treatments; in both treatments, Kraljica showed the highest content (Figure 6B).

Leucine and threonine were significantly elevated in the flour with *Fusarium* inoculation from Tika Taka and Galloper (Figure 6C,D). Tika Taka had the highest increase, of 42% and 44%. In the natural infection group, all varieties had a similar content of leucine, except Vulkan, in which it was significantly decreased, while Kraljica and Galloper had the highest amount of threonine. In the flour with *Fusarium* inoculation, Tika Taka showed the highest content of leucine and threonine, together with Kraljica, which had a high amount of threonine.

### 2.4. Correlation Analysis of Investigated Traits

**Figure 7:**
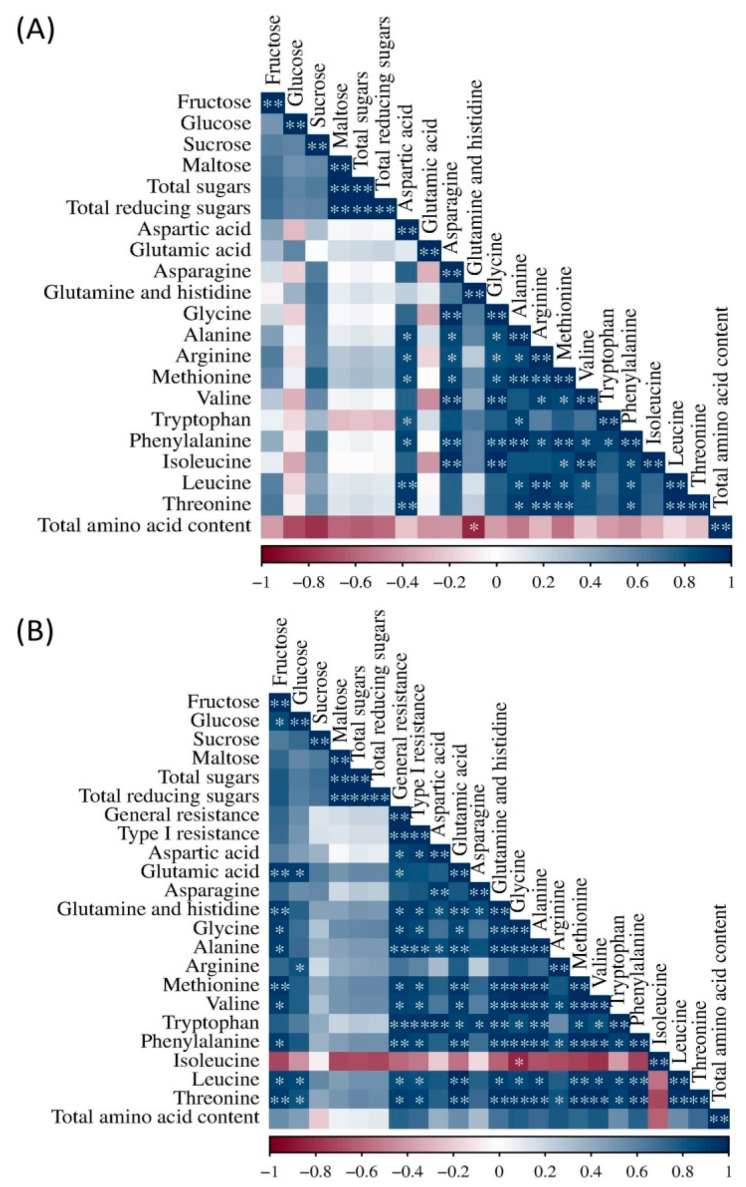
Correlation analysis of the investigated content of sugars and amino acids in flour with natural infection (**A**) and the investigated content of sugars, amino acids, and general and type I *Fusarium* resistance in the *Fusarium* treatment (**B**). *,**—significant correlation at 0.05 and 0.01.

It can be seen that more pronounced correlations were obtained for the flour under *Fusarium* inoculation then for the flour under natural infection (Figure 7A,B). In the natural infection treatment, significant positive correlations were obtained between maltose and total sugars, maltose and total reducing sugars, and total sugars and total reducing sugars (Figure 7A). Amino acids were mostly positively correlated with each other, except glutamic acid, which did not have any significant correlations, and glutamine and histidine, which were negatively correlated with total amino acid content. Also, glutamine and histidine did not significantly correlate with arginine, valine, tryptophan, isoleucine, leucine, threonine, and arginine, and valine did not significantly correlate with tryptophan.

In flour under *Fusarium* inoculation, fructose had significant positive correlations with glucose, glutamic acid, glutamine and histidine, glycine, alanine, methionine, valine, phenylalanine, leucine, and threonine (Figure 7B). Glucose showed highly significant correlations with glutamic acid, arginine, leucine, and threonine. General and type I resistance had significant positive correlations with almost all amino acids. Also, as in natural infection, most amino acids were significantly positively correlated with each other, except isoleucine, which had a significant negative correlation with glycine.

## 3. Discussion

FHB causes significant losses in wheat grain yield because the affected grains are small, shrunken, and of low mass and quality. It is essential to understand the impact of *Fusarium* infestation not only on the properties of wheat grain but also on the properties of the flour that will be used for end-use products. Wheat flour, imparting viscoelastic properties to dough, is used for various purposes of end-use products, including breads, cakes, noodles, crackers, cookies, and pasta [35]. It is known that wheat storage proteins are rich in asparagine, glutamine, arginine, and proline [36], but actual pathogen impact on amino acid and sugar content in the flour is not very clear. Therefore, understanding the defense regulation and function of plants in response to *Fusarium* pathogen attack is desired.

### 3.1. Sugar Changes in Flour with Fusarium Infection

Carbohydrate metabolism can cause various environmental stress responses, as soluble sugars such as sucrose, glucose, and fructose are thought to be involved in the production and regulation of anti-fungal metabolites and in the maintenance of cell homeostasis, but they can also be used as nutrients for pathogen growth [37]. The major carbohydrate found in the endosperm, or flour portion, of the wheat grain is starch; however, other carbohydrates, such as free sugars, glucofructans, and hemicelluloses, can also be found [27]. Carbohydrates account for around 70% of wheat’s composition and are mainly composed of monosaccharides, oligosaccharides, and polysaccharides, and any change in the properties of carbohydrates significantly affects the quality of wheat [38]. The concentrations of soluble monosaccharides and disaccharides in flour are (in decreasing order) as follows: sucrose (2.16 mg/g), maltose (0.57 mg/g), fructose (0.53 mg/g), and glucose (0.45 mg/g) [39]. Very often, FHB infection in wheat results in grain weight loss and carbohydrate and protein composition changes [40]. Research on the impact of *Fusarium* infestation on carbohydrates in wheat flour is scarce, especially on its impact on wheat grains, compared to any other wheat tissue.

According to a study by Žilić et al. [41], within *T. aestivum* species, there was a significant difference in the content of sugars between varieties. In the present study, all varieties, except Tika Taka, which had the most pronounced FHB symptoms, showed a lower variation in the content of sugars between FHB-treated flour and naturally infected flour.

#### 3.1.1. Fructose and Glucose

In the current research study, the most FHB-susceptible variety, Tika Taka, showed a significantly increased average total amount of fructose and glucose in FHB-treated flour compared to naturally infected flour. We can hypothesize that severely infected heads of Tika Taka might have a later ripening of leaves, indicating that assimilates in leaves could not be translocated and utilized by the developing grains. This can be supported by the fact that Tika Taka was the variety with the latest heading and flowering dates. Therefore, these sugars were translocated in the latest stage of grain filling, thus remaining in the grains and flour. For example, under heat stress, the central enzyme in sucrose metabolism, cell wall invertase, catalyzes the irreversible breakdown of sucrose into glucose and fructose and downregulates the genes involved in carbohydrate metabolism [42]. It has been shown that heat and drought stress increases the accumulation of foliar sucrose and decreases starch accumulation [43]. Starch synthase enzymes separate glucose residues from ADP-glucose and bind them to the ends of amylose and amylopectin to elongate polymer polysaccharide chains [44]. Also, it is known that pathogen attack, and ultimately the activation and repression of defense genes related to these processes, are affected by glucose signaling through hexokinase-dependent pathways [45]. Overall, we assume that starch in Tika Taka flour could not be properly produced and therefore glucose remained elevated. Only Vulkan, the most FHB-resistant variety, had a significantly decreased glucose content in FHB-infected flour compared to naturally infected flour. In a previous study, fungal infection with *F. solani* decreased antioxidant activity, total phenolic content, and the contents of protein, sugar (sucrose, fructose, and glucose), and some amino acids (glutamine, serine, and arginine) in roselle (*Hibiscus sabdariffa* L.) seeds [46]. However, in that study, it was not taken into account whether these plants were FHB-susceptible or -resistant, as in the current research study.

#### 3.1.2. Maltose, Total Sugars, and Total Reducing Sugars

Overall, sugars occurring in the wheat endosperm do not play a significant part in the technological quality of wheat flour, especially not in its rheological properties. The only exception could be maltose, as it supposedly forms mostly from the degradation of starch via the activity of α-amylase and β-amylase [47]. Besides that, maltose is mainly derived from the hydrolysis of starch, which occurs due to high α-amylase activity, but the presence of exoenzymes can also cause maltose production [48], as exoenzymes are produced by pathogens that are secreted into the environment and that are capable of degrading insoluble polymers. Maltose content was more than 28 times higher in the naturally infected control grains and was highly affected by the applied fungicide and also by the *Fusarium* susceptibility of wheat varieties. Only the FHB-susceptible Tika Taka variety had a significantly increased average amount of maltose in FHB-treated flour, compared to naturally infected flour.

Sucrose and total sugars were also significantly increased in Kraljica, while Tika Taka had significantly increased total sugars and total reducing sugars. Biemelt and Sonnewald [49] showed that sugars play a key role in plants’ defense responses to infection caused by fungal pathogens. Similarly, as in the current study, the total sugars ranged from 2.29 to 3.82%, while reducing sugars were in the range of 0.97–0.64% in a study on durum wheat [21]. The current research had results with higher values of reducing sugars, as this study was on bread wheat. Overall, total sugars and total reducing sugars were significantly increased in the FHB-susceptible Tika Taka variety, while they did not significantly change in the other varieties.

It is evident from the current study’s results that in the Tika Taka variety, the average total amounts of fructose, glucose, maltose, total sugars, and total reducing sugars were significantly increased in FHB-treated flour compared to naturally infected flour. It seems that other varieties restricted the transfer of sugars through reprogramming carbon metabolism and transport with their host cells [50]. The same researchers concluded that pathogens could repress some transporters to decrease plant defense, resulting in a more beneficial environment for pathogen growth. However, in different plant–pathogen interactions, either a decrease or an increase in the level of sugars in infected tissues was observed [51].

### 3.2. Free Amino Acid Content Fluctuations in Flour with Fusarium Infection

The most dominant amino acid in wheat grains was glutamic acid, with mean concentrations of 30.53–37.18 g/100 g protein [29]. Other amino acids (in grams per 100 g of protein) in flours from the different wheat varieties were as follows: aspartic, 3.28–6.76; serine, 4.17–5.18; glycine, 4.31–7.11; alanine, 5.62–8.70; arginine, 2.10–3.35; tyrosine, 3.03–4.10; cystine, 0.68–4.32; proline, 3.32–14.98; threonine, 0.94–1.96; histidine, 0.82–1.86; valine, 0.82–3.52; methionine, 0.57–2.88; phenylalanine, 2.83–5.56; isoleucine, 2.74–3.77; leucine, 6.31–9.30; and lysine, 1.27–3.61 [29]. Besides sugars, amino acid levels in wheat grains were also related to *Fusarium* infection; for example, alanine, lysine, and tyrosine increased with the percentage of *Fusarium*-damaged grains or DON contents, whereas glutamic acid contents decreased [52]. Also, the research of Pratelli and Pilot [53] suggested that pathogenic infection resulted in many changes in amino acid metabolism expression.

#### 3.2.1. Non-Essential Amino Acids in FHB-Susceptible Varieties

Non-essential amino acids, comprising aspartic acid, glutamic acid, serine, glycine, alanine, arginine, tyrosine, cysteine, and proline, constitute 65.85–76.19% of the total amino acids. They are related to gluten proteins (gliadin and glutenin) and have an important role in the end-use product of wheat flour [29]. In the current research, it was evident that the two most FHB-susceptible wheat varieties (El Nino and Tika Taka) had significantly increased free aspartic acid, glutamic acid, glycine, alanine, arginine, and asparagine contents in FHB-infected flour compared to naturally infected flour.

Aspartic proteases have the presence of two aspartic acid residues located within the conserved Asp-Thr/Ser-Gly motif, responsible for catalytic activity [54], and therefore, by increasing protease activity via *Fusarium* spp. in two FHB-susceptible varieties, aspartic acid was also increased. Further, glutamic acid could increase the virulence of *Fusarium* spp., as was observed in a study by Li et al. [55], where glutamic acid increased the virulence of *Vibrio splendidus* via an increase in bacterial motility. Due to the significant increases in free glycine and alanine in three varieties (two out of three—El Nino and Tika Taka—were very FHB-susceptible), it might be concluded that these were amino acids that wheat plants produce to defend themselves. In one study, the activity of *Bacillus subtilis* was inhibited by multiple end products of glutamine metabolism (e.g., AMP, alanine, glycine) [56]. For arginine, it was observed that it promotes plant photosynthetic capacity and alters amino acid metabolism [57]. Therefore, in the current research study, some varieties (such as the most FHB-susceptible Tika Taka) were still trying to defend themselves by activating amino acid metabolism. The two most FHB-susceptible varieties (Tika Taka and El Nino) had a significantly elevated asparagine content in FHB-infected flour. Asparagine accumulates during abiotic and biotic stress and has a role in reactive oxygen species/nitrous oxide production [58]. In addition, free asparagine is the main precursor of acrylamide, a thermally induced contaminant in food. According to the results of Žilić et al. [59], a high correlation (r^2^ = 0.99) between free asparagine and acrylamide was found in whole-grain bread wheat-based biscuits. The asparagine alone may be thermally converted into acrylamide, but the yield of acrylamide from asparagine is much higher when a carbonyl source (principally glucose, fructose, and maltose) is present [60]. In this regard, previous results also showed the influence of starch damage in flour on the increase in acrylamide content in thermally treated foods [61]. Therefore, winter wheat breeders may have an opportunity to reduce free asparagine concentration in the grain by identifying and selecting genotypes carrying FHB resistance.

#### 3.2.2. Essential Amino Acids in FHB-Susceptible Varieties

Essential amino acids, which include threonine, histidine, valine, methionine, phenylalanine, isoleucine, leucine, and lysine, account for 19.13–27.43% of the total amino acids. Essential amino acids such as histidine (plus non-essential glutamine) and tryptophan were significantly elevated in Tika Taka and El Nino (the most two FHB-susceptible varieties), while methionine, phenylalanine, leucine, and threonine were only elevated in Tika Taka, along with methionine, phenylalanine, leucine, and threonine in Galloper. The increase in these amino acids may be due to an interference in host metabolism by the pathogen or due to host–pathogen interaction. An increment in amino acids was also noticed during the fermentation process of bread and could be explained by the fact that during fermentation, carbohydrates serve as an energy source for fungal growth, and some of them may be converted into protein [62]. Further, it can be hypothesized, according to the results of the current study, that in more FHB-susceptible varieties, high concentrations of specific amino acids were mostly secreted by pathogens. Cuperlovic-Culf et al. [63] reported that in susceptible varieties, increases in amino acid content may be the result of fungal metabolism.

Histidine is particularly important in the context of protein content, as it was discovered that a certain correlation may exist between QTLs controlling protein content and histidine content in barley grains [64]. Zhou et al. [65] reported that grain weight and starch and protein content increased with increasing glutamine concentration. It was seen in the current research that glutamine and histidine were significantly decreased only in the most FHB-resistant variety (Vulkan) in FHB-treated flour. A previous study by Li et al. [66] showed that FHB resistance resulted from a rare deletion involving the 3′ exon of the histidine-rich calcium-binding protein gene on chromosome 3BS. In the current study, the content of methionine was increased in two varieties (one, Tika Taka, was very susceptible), and it was previously reported that changes in methionine content contribute to the ability of the plant to manage stress [67]. One study showed that some *Fusarium* spp. excrete high levels of valine [68]. This might also have occurred in the current research, in which the most FHB-susceptible variety (Tika Taka) had the highest valine content, with a significant increase in flour under the FHB treatment.

FHB infection in the model cereal species *Brachypodium distachyon* resulted in an increased production of tryptophan-derived metabolites, as well as of phenylpropanoids and phenolamines [69]. In the current research, the highest level of leucine in FHB-treated flour was found in the most susceptible variety. It has been reported that the contribution of wheat-specific leucine-rich repeat receptors like kinase homologs has a relation to *Fusarium* resistance [70]. However, it was seen that isoleucine is the lowest in FHB-susceptible and FHB-resistant varieties. The amino acids threonine and isoleucine are actually the most important in animal nutrition [71], and in the current research, it can be seen that the amount of isoleucine was variety-dependent. Threonine is derived from the aspartate pathway, and the amino acid imbalance-associated threonine accumulation was found to increase plant immunity to some pathogens [72].

#### 3.2.3. Amino Acid Content in Relation to FHB Resistance

On the other hand, the most FHB-resistant variety, Vulkan, showed a significant decrease in phenylalanine and glutamine plus histidine in FHB-infected flour compared to naturally infected flour. The decrease in amino acids in the infected tissues may be due to their utilization by the pathogen or to their utilization in the synthesis of proteins during host–parasite interactions. Previously, one study showed that phenylalanine and malate exhibited a strong inhibiting effect on *F. graminearum* growth in wheat spikelets harvested on day 4 after *Fusarium* inoculation [73]. They could be used as a green fungistatic agent in resistance to FHB. It has been reported that glutamine has also been associated with fungal hyphal growth [74]. Glutamine synthetase (GS) plays a major role in plant nitrogen metabolism, but the roles of individual GS isoforms in grains are unknown [75]. GS is an enzyme that plays an essential role in the metabolism of nitrogen by catalyzing the condensation of glutamate and ammonia to form glutamine. Glutamic acid is an amino acid that is between the free forms of glutamine within the building blocks of protein. It is converted to glutamine by attaching to a mineral ion. Probably, more resistant varieties had the fastest and strongest response, primarily through the activation of metabolites produced in different stages but much earlier in the growing season [9,76] than those investigated in mature grains in the current study. Therefore, the decreased content of phenylalanine and glutamine plus histidine may be related to FHB resistance.

### 3.3. Correlation of FHB Resistance with Sugar and Free Amino Acid Content

The results of the current research showed differences in the amino acid composition of flours from different wheat varieties under two treatments (natural infection and *Fusarium* inoculation). Siddiqi et al. [29] reported that the amino acid composition in flours from different wheat varieties was the result of growing environmental conditions like CO_2_ concentration and temperature, wheat types, the protein content of the flour, the extraction rate, and the application of fertilizers. Besides that, the days after *Fusarium* inoculation influenced the levels of amino acids in the two varieties, and the changes in amino acids were related to FHB [33].

In general, a review by Morkunas and Ratajczak [77] reported a strong correlation between soluble sugar concentration and stress tolerance in plants. Due to the increased total sugars and total reducing sugars in Tika Taka, we might conclude that other varieties (except Kraljica for total sugars) limited pathogen access to nutrients and initiated immune responses. Overall, in the current study, it was evident that a high sugar level stimulated the development of the pathogenic fungi. Some fungi have the enzyme invertase as the constituent that catalyzes sucrose hydrolysis. Also, it is important to note that glucose and fructose are produced by the hydrolysis of sucrose [78], which might be hydrolyzed by *Fusarium* fungi [79]. In flour under the *Fusarium* treatment, both fructose and glucose showed significant correlations with specific amino acids. However, the content of fructose was correlated with more amino acids, showing a larger influence of that sugar on plant FHB resistance or susceptibility.

Correlation analysis led to the hypothesis that in resistant varieties, branched-chain amino acids are produced by wheat and in susceptible varieties, they might be the result of fungal metabolism [63]. This hypothesis can also be applied to the current work, where it is clearly visible that more sensitive varieties have an increase in amino acid content. Also, previous studies have shown that regulating the metabolism of a certain amino acid affects the level of other amino acids, as the biosynthesis and catabolism of amino acids are derived from the same metabolic trunk and/or are closely related to other metabolic pathways [71]. Another finding showed that wheat varieties express a high genetic variability and selection possibility for gentiobiose, butyric acid, galactopyranosyl, phenylalanine, tryptophan, leucine, and isoleucine [80].

For example, in the current study, flour under FHB treatment contained isoleucine that was negatively correlated with glutamine and histidine, compared to the flour under natural infection, where this correlation was not significant. It has been reported that isoleucine is a branched-chain amino acid that can also be induced by the aspartate pathway, and it could have a major role in plant stress resistance [81]. Further, the host methionine cycle is associated with the transmethylation and metabolism of amino acids such as serine, glycine, glutathione, and taurine [82]. Overall, amino acids could have a major role in signaling processes at the beginning of the infection process, which would strengthen plant defenses to effectively resist pathogenic attacks. In more FHB-susceptible varieties, this was not possible, after which the pathogen started to produce some amino acids in the wheat grains.

The increased general and type I susceptibility indicate an amino acid synthesis altered by pathogens. It seems that sugar and amino acid metabolism closely interacts with FHB synthesis in highly FHB-sensitive varieties, and the results give important additional information on the altered metabolism of FHB-attacked plants. This was already concluded by Zhao et al. [28], who reported that some of the amino acid contents changed greatly in varieties with different FHB resistance levels when infected with *F. graminearum*.

## 4. Materials and Methods

### 4.1. Field Experiment

**Figure 8:**
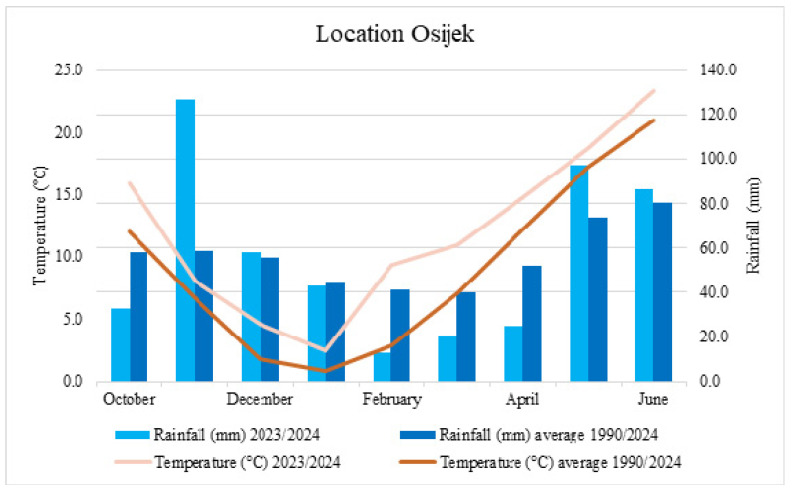
Total monthly rainfall (mm) and average temperature (°C) during the growing season from October 2023 to June 2024, as well as during the growing season for the period 1990–2024 taken as the multi-year average.

Field research was conducted during 2023–2024 with six winter wheat varieties (Golubica, Tika Taka, El Nino, Galloper, Kraljica, and Vulkan) produced by Agricultural Institute Osijek (Croatia). These varieties vary from being resistant to very susceptible to FHB. The experiment was conducted using two treatment combinations in a randomized complete block design with two replications. The field research was conducted at the Agricultural Institute Osijek, Croatia (45°27′ N, 18°48′ E), on eutric cambisol (pHKCl–6.25, humus–2.00–2.20%). Each individual plot was 7 m long and 1.08 m wide, and seeds were planted with a Hege Seedmatic machine in October 2023. As a control of seed-borne diseases, MAXIM^®^ (Fludioxonil) was used at a rate of 125 mL 100 kg^−1^. The agro-technical practice used in this experiment was standard for commercial wheat production, except for fungicide application, which was omitted. In February 2024, plants were treated by the herbicide Sekator (100 g/L amidosulfuron and 25 g/L iodosulfuronmethyl-sodium in 1 L, Bayer) in the amount of 0.15 L/ha and Nuance (tribenuron-methyl, Nufarm) in the amount of 12 g/ha, while protection was again performed in March with Cyclone (sulcotrione 250 g/L, VillaCrop) in the amount of 0.2 L/ha, Nuance in the amount of 12 g/ha, and Bonaca (fluroxypyr-meptyl, Galenika-Fitofarmacija a.d.) in the amount of 0.5 L/ha. The experiment included two different treatments (*Fusarium* inoculation and untreated control). Plant density was 3000–3500 plants per 7.56 m^2^. The annual precipitation during the growing season 2023/2024 was 502.1 and the average annual temperature was 12.0 °C (Figure 8). Data were obtained from the Croatian Meteorological and Hydrological Service.

### 4.2. Inoculum Production and Inoculation

Two *Fusarium* species were used for inoculum production: *F. graminearum* (PIO 31), previously isolated from winter wheat collected in East Croatia, and *F. culmorum* (IFA 104) obtained from IFA-Tulln, Tulln, Austria. The conidial inoculum of *F. graminearum* and *F. culmorum* was produced by a mixture of wheat and oat grains [83]. For the mass production of the conidia of each isolate in a proportion of 1:1., two Fdisks (5 mm diam.) from a well-grown colony were transferred to the mixture of wheat and oat (3:1) in glass jars, previously soaked in water overnight, and autoclaved. The glass jars were then left at room temperature and in diffused daylight and were shaken daily for two weeks to ensure proper aeration and drying. Macroconidia were washed off the colonized grains, and the suspension was diluted for inoculation. The final conidial concentrations of both species were determined using a hemocytometer (Bürker-Türk, Hecht Assistent, Sondheim vor der Rhön, Germany) and were set to 1 × 10^5^ mL^−1^. The spore suspensions were set to a concentration so that a single bottle of one strain contained a sufficient amount of the suspension (>900 mL) so that it could be diluted in 100 L of water immediately before inoculation (100 mL/m^2^). One treatment was grown according to standard agronomical practice with no usage of fungicide and without misting treatment, while another treatment was subjected to two inoculation events using a back sprayer with the spores of two *Fusarium* spp prepared earlier at the time of flowering (Zadok’s scale 65) [84]. Inoculations were performed on the whole plot area, which consisted of 4400–4600 plants, in two replications. Misting was provided by spraying with a tractor back-sprayer on several occasions to provide moisture for the development of the infection.

### 4.3. Disease Assessment

General (disease severity) and type I resistance to FHB (resistance to initial infection within the spike) were evaluated at 10, 14, 18, 22, and 26 days after inoculation. Disease severity (general resistance) was estimated based on the percentage of bleached spikelets per plot according to a linear scale (0–100%), while disease incidence (type I resistance) was calculated as the percentage of diseased ears after assessing a random sample of 30 heads. All these values were used to calculate the area under the disease progress curve (AUDPC) [85].

### 4.4. Analytical Procedures

White flour was produced from each sample by laboratory-scale milling using a Quadrumat mill (Brabender OHG, Duisburg, Germany).

#### 4.4.1. Analysis of Sugars

The analysis of sugars was carried out according to the method described previously [38]. Briefly, the analysis of extracted sugars was performed on a Thermo Scientific UltiMate 3000 high-performance liquid chromatography (HPLC) system using a refractive index detector (RID) and isocratic elution with a mobile phase consisting of 80% ACN in water at a flow rate of 0.7 mL/min. A Thermo Scientific Hypersil GOLD Amino column (150 mm × 3 mm with 3 µm diameter particles) was used for sugar separation. Fructose, glucose, sucrose, and maltose peaks were identified according to standard retention times and quantified using external calibration curves. The sugar contents were expressed as the percentage of sugar per dry matter (d.m.).

#### 4.4.2. Analysis of Free Amino Acids

Free amino acids from wheat flour were extracted with water for 1h. After shaking and centrifugation, amino acid extracts were filtered through a 0.45 μm nylon syringe filter and prepared for HPLC analysis. The derivatization of amino acids was performed before the analysis using a slightly modified o-phthaldialdehyde (OPA) solution [86]. The HPLC analysis was carried out with a Thermo Scientific UltiMate 3000 HPLC system using a diode array detector (DAD) and a linear gradient elution program with a mobile phase containing solvent A (40 mM Na_2_HPO_4_, pH 7.8) and solvent B (acetonitrile/methanol/water, 46:46:10, *v*/*v*/*v*). A Thermo Scientific Hypersil GOLD Amino C18 column (250 mm × 4.6 mm, 5 μm) was used for amino acid separation according to the procedure defined by Sarić et al. [87]. The identified amino acid peaks were confirmed by Thermo Scientific Dionex Chromeleon 7.2 chromatographic software according to standard retention times and were quantified using external calibration curves. The results are expressed as mg of amino acid per kg of d.m.

### 4.5. Statistical Analysis

A total number of four sets of pooled flour samples, each derived from at least two different replications per genotype, were used for the carbohydrate analyses, i.e., the data are based on four biological replications. For amino acid analysis, two biological replications per treatment represented the mean values for each of the varieties.

Analysis of variance (ANOVA) was conducted using a main-effects model, and relative differences in grain sugar and amino acid properties between FHB-inoculated and naturally infected samples were analyzed for statistically significant differences by Fisher’s least significant difference (LSD) test (α = 0.05) using Statistica version 13.3 (Statsoft Inc., Tulsa, OK, USA). The LSD value was used to evaluate whether the difference in performance observed between treatments for each variety and for all varieties separately in one treatment (naturally infected flour vs. flour from inoculated plants) was significant. Error bars were used to represent standard errors.

## 5. Conclusions

A severe *Fusarium* attack can lead to changes in the metabolism of plants and can decrease the incorporation of sugars in starch production; therefore, a part of the sugars remains in the leaves and the young developing grains. It can be assumed that *Fusarium* spp. utilize sugars, as well as that the most susceptible variety, Tika Taka, did not restrict the amount of sugars in grains, allowing the pathogen to spread severely. However, the total level of sugars in FHB-infected flour, as well as the proportions of individual sugars, may differ as a result of regulatory mechanisms in plants and due to pathogen interference. On the other hand, a decreased content of phenylalanine and glutamine plus histidine may be related to FHB resistance. Based on previous research and data in the literature, it could be assumed that major changes in the concentration of amino acids in more FHB-resistant varieties are the product of plant metabolism, while the high concentrations of amino acids in FHB-susceptible varieties are secreted by the pathogen. We can conclude that there is a risk of FHB-susceptible varieties forming acrylamide in thermally processed wheat-based foods due to free asparagine and sugars, which were significantly elevated in infected flour. Therefore, the results can have also practical significance beyond the scientific findings of the article and may serve as a starting position for further research. This is particularly important in the context of public health and food safety, as mycotoxins that have detrimental effects on the quality and processing performance of wheat are also developed by *Fusarium* spp.

## Data Availability

Data are contained within the article.
